# Genetic parameters for marbling and body score in Anglonubian goats using Bayesian inference via threshold and linear models

**DOI:** 10.5713/ajas.17.0490

**Published:** 2018-03-02

**Authors:** Luiz Antonio Silva Figueiredo Filho, José Lindenberg Rocha Sarmento, José Elivalto Guimarães Campelo, Marcos Jacob de Oliveira Almeida, Antônio de Sousa, Natanael Pereira da Silva Santos, Márcio da Silva Costa, Tatiana Saraiva Torres, Luciano Silva Sena

**Affiliations:** 1 Federal Institute of Education, Science and Tecnology of Maranhão (IFMA), Caxias, MA 65-468, Brazil; 2 Federal University of Piauí (UFPI), Teresina, PI 64049-550, Brazil; 3 Brazilian Agricultural Research Corporation (Embrapa Meio Norte), Teresina, PI 64006-220, Brazil; 4 Technical School of Teresina (CTT), Teresina, PI 64049-550, Brazil; 5 Federal University of Piauí (UFPI), Bom Jesus, PI 64900-000, Brazil

**Keywords:** Bayesian Inference, Carcass, Components of (co) Variance, Gibbs Sampling, Heritability

## Abstract

**Objective:**

The aim of this study was to estimate (co) variance components and genetic parameters for categorical carcass traits using Bayesian inference via mixed linear and threshold animal models in Anglonubian goats.

**Methods:**

Data were obtained from Anglonubian goats reared in the Brazilian Mid-North region. The traits in study were body condition score, marbling in the rib eye, ribeye area, fat thickness of the sternum, hip height, leg perimeter, and body weight. The numerator relationship matrix contained information from 793 animals. The single- and two-trait analyses were performed to estimate (co) variance components and genetic parameters via linear and threshold animal models. For estimation of genetic parameters, chains with 2 and 4 million cycles were tested. An 1,000,000-cycle initial burn-in was considered with values taken every 250 cycles, in a total of 4,000 samples. Convergence was monitored by Geweke criteria and Monte Carlo error chain.

**Results:**

Threshold model best fits categorical data since it is more efficient to detect genetic variability. In two-trait analysis the contribution of the increase in information and the correlations between traits contributed to increase the estimated values for (co) variance components and heritability, in comparison to single-trait analysis. Heritability estimates for the study traits were from low to moderate magnitude.

**Conclusion:**

Direct selection of the continuous distribution of traits such as thickness sternal fat and hip height allows obtaining the indirect selection for marbling of ribeye.

## INTRODUCTION

In animal breeding programs several traits are evaluated to identify genotypes with higher average according to the proposed objectives. The analyzed data for this purpose may be continuous, as an example of measurement performed in the loin eye area, which is assumed to have normal distribution, or discontinuous such as the case of categorical traits as carcass marbling. However, to obtain estimates of (co) variance components and genetic parameters for categorical data, some approaches that consider the discrete data distribution should be used to ensure greater accuracy of the estimates.

Within the categorical traits in meat goat production, the score assigned in the evaluation of body condition of animals is a criterion that has been used for carcass *in vivo* evaluation, whose advantage is to obtain information of the nutritional status of animals. In goats, this technique is widely applied, however the use of ultrasound to evaluate carcass in live animals has spread because it enables the identification of carcass marbling based on intramuscular fat. It is a type of data with discrete distribution in which the *longissimus dorsi* is the main muscle for evaluation in the living animal.

The estimation method with establishment of a model that correctly describes the nature of categorical data is an important factor to obtain genetic parameters [1]. The methodology of Mixed Linear Models is the most common means of estimating (co) variance components for traits of economic interest since this methodology has easy application in animal model and requires less time in data processing. However, it is necessary that the analyzed traits have normal distribution, which is noticed only in continuous data. Thus, the use of important characteristics for animal breeding which assume discrete distribution is limited. Genetic analyses would not be appropriate using linear models [2,3]. Therefore, the use of threshold models is recommended, because they are more efficient in detecting genetic variability, in comparison to linear models [3].

With the use of threshold model it is considered there is an unobservable variable that takes the normal distribution underlying the discrete variable. The variable connection observed with the underlying continuous scale is made by a set of fixed thresholds. Thus, the underlying variable, defined as the sum of genetic and environmental effects which affect the susceptibility of a trait [2], is described by a linear model, but the relationship of this variable to the observed scale is non-linear [4,5].

By comparison, the genetic gain estimated from genetic analysis of categorical data obtained by threshold models are higher due to the achievement of higher heritability in the underlying scale [6], thus resulting in better identification of higher value genotypes.

With the advent of increasingly powerful processors, the Bayesian methodology reemerged as statistical tool to estimate components of (co) variance and genetic parameters in animal breeding. Bayesian inference allows the use of prior information of the studied trait being included in the analysis through information of a prior distribution of the parameters to be analyzed along with its uncertainty before the observation data. It also considers the different distributions of the studied data, increasing the accuracy of estimates and predictions.

The aim of this study was to estimate (co) variance components and genetic parameters for categorical carcass traits using Bayesian inference via mixed linear and threshold animal models in Anglonubian goats reared in the Mid-North region of Brazil.

## MATERIALS AND METHODS

The experimental procedure was approved by the Institutional Animal Care and Use Committee at Federal University of Piauí, Brazil (009/14).

The database used in this research consisted of genealogical and production information measured in registered Anglonubian goats with genealogical records reared in the states of Piauí and Maranhão (Mid-North region of Brazil). The field data collection was carried out from the years 2012 to 2014.

The traits evaluated in this study were: body condition score (BCS), marbling in the rib eye (MRE), ribeye area (REA), fat thickness of the sternum (FTS), hip height (HH), leg perimeter (LP), and body weight. These traits were measured at the same time in each animal. Goats of both sexes, aged from seven months and healthy were eligible to participate.

For standardization during the farms data collection the following steps were adopted: after weighing on a scale with a capacity of 200 kg and precision of 0.10 kg, BCS of each animal was measured based on palpation and visualization of the lower back region, mimicking up motion grippers applying constant pressure around and between the transverse and spinal apophyses, also in the sternal region, assessing the amount of skin, muscle and fat density in two anatomical regions, according to methodology cited by [7]. The score assigned for each individual was based on the perception of the fat and muscle deposition in the evaluated areas, taking as a base values from one (lowest fat deposition) to five (excessive deposition of fat).

Thereafter, the following morphometric measurements were taken using hipometer and measure tape while the animal was restrained in a comfortable standing position: HH, distance between the sacral tuberosity of the ilium and the soil; and LP, measured on the median part of the leg above the femoral-tibial-rotulian articulation (in centimeters).

*In vivo* evaluation of carcass was performed by means of ultrasound images captured using the apparatus Chison 600M equipped with linear transducer (13 cm) using a set of frequency 5.0 MHz.

The REA (in cm^2^) was measured through ultrasonographic cross-sectional images of the *longissimus dorsi* muscle in the intercostal space between the 12th and 13th ribs. With the same image the MRE was evaluated with by assigning grades from 0 (absence of intramuscular fat) to 6 points (abundance of intramuscular fat). This visual grading scale was an adaptation of that used by [8].

The subcutaneous FTS, given in mm, proposed by [9] to indicate carcass fat thickness was measured from ultrasound images of mediastinal sternal region (3rd bone of the sternum). During ultrasound readings animals were restrained, in order to keep their comfort for better quality images.

In the statistical analyses it was assumed that the BCS and MRE data follow discrete distribution while REA, FTS, HH, and LP follow continuous distribution, so that these traits were considered anchors in two-trait analyses with the two categorical traits.

Information of months in which data were collected was grouped in two collection seasons (CS): rainy season, from January to May; and dry season, from June to December. The birth month of animal was also distributed in two birth seasons, namely rainy and dry seasons, similar to the format of CS. The age of the animal at the time of collection was grouped into age classes (AC), as follows: AC = 1, animals >six months and <two years; AC = 2, animals >two years but <four years; AC = 3, animals >four years. Finally, the evaluated goats were grouped into animal category (CAT) as follows: pregnant, kidding, and dry breeding does. In the formation of the contemporary group (CG), animals born on the same farm, in the same year, and same sex were taken into consideration.

The data were edited and formatted with the statistical program SAS (SAS Inst. Inc., Cary, NC, USA). After the consistency analyses a file containing animal information, parent, CG, year of collection (YC), age group (AC), animal category (CAT), and observations relating to the analyzed traits, totaling 385 animals with observations was edited. At the end, the numerator relationship matrix of Wright contained information from 793 different animals.

The components of (co) variance and genetic parameters were estimated via linear and threshold animal model by Bayesian inference in single and two-trait analyses. Two-trait analyses were performed by combining the two categorical traits (two by two) with the continuous ones, totaling 11 analyses. Thus, two-trait analyses were performed in animal model, assuming two different distributions in the same analysis.

Estimates of genetic parameters via Bayesian Inference were calculated with the aid of GIBBS1F90 and THRGIBBS1F90 applications, used for linear and threshold models, respectively [10], by testing chains with 2 and 4 million cycles. The length of the chain used to compare models and generate the posterior distribution of the (co) variance components and genetic parameters of the BCS and MRE was 2 million, since both generated similar estimates. This similarity was considered as the criterion of convergence.

After burn-in of the first 1,000,000 samples samples were taken apart at every 250 cycles (sampling interval), resulting in *a posteriori* distribution with 4,000 samples in which inferences were performed. Values for burn-in and sampling interval were defined based on preliminary analyses in which the convergence and distribution of samples were evaluated through the POSTGIBBS1F90 program [10], which uses the Geweke diagnostic test [11] based on the Z test of average equality of the conditional distribution data logarithm.

The animal model in matrix notation was: *y* = *Xβ*+*Zα*+*ɛ*, in which: *y* = vector of observations of the studied traits (underlying scales for categorical traits); ***X*** = matrix n×f of incidence (n = total of observations, and f the number of fixed effects of classes), which relates the findings to the systemic effects; *β* = vector of systemic effects of CG (formed by animals raised on the same farm, born in the same year and season, and evaluated in the same season), YC, AC, and CAT; ***Z*** = the matrix n×N of incidence, which lists the observations to genetic additive direct effects, where n is the total number of observations and N number of individuals; *α* = vector of random effects representing the direct genetic additive values for each animal (animal model); and *e* = vector of residual random errors associated to the observations.

In Bayesian analysis the systemic and random effects included in the model are considered as random variables.

The accepted assumptions, with a focus on Bayesian methodology, about the information (*y*) and data (*β*, *α*, and $\sigma_{e}^{2}$), which assumes multivariate normal distribution, is represented as: *y*|*β,α*, $\sigma_{e}^{2}\sim N(X\beta +Z\alpha +I\sigma_{e}^{2})$; *α*|*A,* $\sigma_{a}^{2}\sim N(0,A\sigma_{a}^{2})$;*e*|*e,* $\sigma_{e}^{2}\sim N(0,I\sigma_{e}^{2})$; thus: $\sigma_{a}^{2}$ and $\sigma_{e}^{2}$, components of additive direct genetic and residual (co) variance, respectively; ***A***, numerators matrix of Wright’s inbreeding coefficient; and ***I***, identity matrix of equal order to the number of animals with observations.

In the threshold model the underlying scale assumes normal distribution represented as: $U|\theta \sim N(W\theta ,I\sigma_{e}^{2})$ in which *U* is a vector of scale base of origin *r*; *θ* = (*b*, *a*) is the location vector of order parameters *s* with *b* (fixed effects in frequentist analysis) and order *s* with *a* (random genetic additive direct effect); ***W*** is a matrix incidence of order *r* by *s*; ***I*** is an identity matrix of order r by *r*; and $\sigma_{e}^{2}$ is the residual variance.

The conditional probability that *y* *_i_* that falls into the category *j* (1, 2, 3, 4, 5: BCS; and 1, 2, 3, 4, 5, 6: MRE) with the given vectors *β*, *α* and *t* (*t* = *t* *_min_*, *t* *_1_*, ..., *t* *_j-1_*, *t* *_máx_*) and represented as:

$$\begin{array}{l} \text{Pr}(y_{i}=j|\beta ,a,t)=\text{Pr}\left(t_{j-1}<U_{i}<t_{j}|\beta ,a,t\right)\\ =\Phi \left(t_{j}-{X^{\prime }}_{i}\beta -{z^{\prime }}_{i}a-{z^{\prime }}_{i}c\right)-\Phi \left(t_{j-1}-{X^{\prime }}_{i}\beta -{z^{\prime }}_{i}a-{z^{\prime }}_{i}c\right)\\ =p(y_{i}|\beta ,a,t). \end{array}$$

The categories (scores) of the BCS and MRE traits for each *i* animals are defined by *Ui*, in the underlying scale, and BCS:

$$\begin{array}{l} y_{i}=\{\begin{array}{l} 1\;t_{0}<U_{i}\leq t_{1}\\ 2\;t_{1}<U_{i}\leq t_{2}\\ 3\;t_{2}<U_{i}\leq t_{3}\\ 4\;t_{3}<U_{i}\leq t_{4}\\ 5\;t_{4}<U_{i}\leq t_{5} \end{array}\;i=1,\ldots ,n,\text{and}\\ \text{for MRE:}\;y_{i}=\{\begin{array}{l} 1\;t_{0}<U_{i}\leq t_{1}\\ 2\;t_{1}<U_{i}\leq t_{2}\\ 3\;t_{2}<U_{i}\leq t_{3}\\ 4\;t_{3}<U_{i}\leq t_{4}\\ 5\;t_{4}<U_{i}\leq t_{5}\\ 6\;t_{5}<U_{i}\leq t_{6} \end{array}\;i=1,\ldots ,n, \end{array}$$

Where *n* is the number of observations for each trait.

As the residual variance is not estimable in threshold models, the parameter was assigned a value of 1 in order to obtain identifiability in the likelihood function [12]. This assumption is standard in categorical data analysis.

Flat was defined (uninformative *a priori* distribution) for all initial variance, in other words, did not reflect the knowledge about the parameters to be estimated.

As the best model selection criteria for the traits in study it was used: deviance information criterion (DIC), which is obtained based on the posterior distribution of the deviation statistic [13]; and bayes factor (BF), which is the reason of marginal likelihoods between two models [14].

DIC is a model comparison criterion following the proposition of [15], who suggests that comparisons among models are based on *a posteriori* distributions of the deviance of each model, being defined by: DIC = *D̄* (*θ*) + *p* *_D_*; in which: *D̄* (*θ*) is the global adjustment measure that is the *posteriori* average of the *deviance*; *p* *_D_* is the penalty for complexity of the model (effective number of parameters) given by the difference between the average *posteriori* of the *deviance* and the *deviance* of the averages a *posteriori* of the model parameters of interest. Thus, the smaller the value of DIC the better the fit of the evaluated model [13]. As for FB, the marginal likelihood of a given model M is given by: *f*(*y*|*M*) = *∫ L*(*θ*|*y*) *π* (*θ*) *dθ*

In analysis with two models (*M* *_i_* and *M* *_j_*), the BF was defined as the reason for the marginal likelihoods of these two models: $FB_{i,j}=\frac{\left[f(M_{i}|x)/f(M_{j}|x)\right]}{\left[f(M_{i})/f(M_{j})\right]}=\frac{f(y|M_{i})}{f(y|M_{j})}$.

The interpretation of this ratio was given according to [14], where: *FB* *_i,j_*>1 is the indication that the numerator of the model (*M* *_i_*) is the most plausible if *FB* *_i,j_*<1, the denominator model (*M* *_j_*), is preferred, and if *FB* *_i,j_* = 1, the quality of the two models is the same (*M* *_i_* = *M* *_j_*). The threshold model was the FB denominator.

In two-trait analyses it was assumed normal distribution traits for REA, FTS, HH, LP, and body weight, whereas for MRE and BCS these traits were considered as categorical discrete distribution. The same animal model of single trait was used in the analyses with the following presupposition: *y*|*β*, *a*, *G*_0_, *R*_0_~*N* (*Xβ*+*Za*, *R*_0_⊗*I*); *a*|*G*_0_, *A*~*N*(0, *G*_0_ ⊗*A*); *e*|*R*_0_ ~*N*(0, *R*_0_ ⊗*I*), thus: *A*, additive genetic relationship matrix among animals; *G*_0_, additive genetic (co) variance matrix among traits; *I*, identity matrix; and *R*_0_, residual (co) variance matrix among traits.

Convergence was monitored by Geweke criteria [11] and error of Monte Carlo chain, which was obtained by calculating the variance of samples for each component divided by the number of samples. Thus, the square root of this value refers to the approach of the error standard deviation associated with the size of Gibbs chain [16].

## RESULTS

Table 1 shows the descriptive statistics of MRE and BCS traits. For the other characteristics considered as “anchors” in multi-trait analysis descriptive statistics were presented in [17]. Because they are categorical qualitative data, MRE and BCS were represented more efficiently by the central tendency statistics, namely Median and Mode, which showed value 3.

In the examination of chain convergence, the Geweke criterion was significant (p<0.05) (Table 2), indicating that the chains of the converged parameters and the number of iterations were appropriate, thus validating the estimates of *a posteriori* distribution parameters, according to [11].

Comparing the linear model with the threshold applied to categorical data, using the DIC criteria, and the BF, it was observed that the threshold DIC model showed values equal to 421.00 and 706.74 (Table 2), whereas the linear model presented 956.44 and 1,176.10 for BCS and MRE, respectively. Thus, as the values of the threshold model of DIC were lower than those for linear model, this indicates that it was the best model to fit the data in study, consequently the most recommended model for obtaining estimates of genetic parameters for BCS and MRE in goats.

BF, according to the used criterion, showed values lower than 1, since the denominator model was the threshold, then confirming that it is the most recommended for the inference of parameters, as interpretation proposed by [14].

Estimates of heritability for BCS and MRE traits (0.11 and 0.03, respectively) obtained by the threshold model were lower than the values estimated by linear model. BCS equals to 0.29 and MRE equals to 0.12. The confidence intervals of heritability estimates showed variation of low magnitude when estimated by the threshold model. This amplitude permits inference with a greater reliability of the quality of the estimates.

In two-trait analyses, where the marbling and BCS were paired with other traits with continuous distribution, it was used the threshold model for the categories that were the best adjustment for the ones used in single-trait analysis, while for continuous distribution the model was linear (Table 2).

The estimated heritability for continuous traits ranged from moderate (0.24) to high magnitude (0.54) (Table 3). Table 4 provides estimates of genetic correlations of the categorical traits BCS and MRE with those considered “anchor” in two-trait analyses (REA, FTS, HH, LP, and body weight), in which values were positive and had low to moderate magnitude with the exception of the association between BCS and HH equals zero.

## DISCUSSION

The good availability of food for the evaluated animals in study is well portrayed by the amount of body condition presented, in which the average was 3.27, higher than the median and mode. Therefore, animals with good tissue composition in terms of muscle mass in the lower back were samped. It is noteworthy, however, that animals which participated in exhibitions at agricultural fairs were also sampled, and this partly explains the relatively high value of the observed variation coefficient.

In addition, since BCS and MRE are evaluated subjectively, these traits are greatly influenced by the experience of the appraiser and environmental factors such as food, physiological status, and health, which in this study are embedded in the value of CV 45.71 and 34.11 for BCS and MRE, respectively, enhancing the role of phenotypic variability that should be considered carefully if included in animal breeding programs.

For the carcass fat thickness based on the value of MRE, the average of 2.55 points, that median and mode corrected to 3.0, is greater than what was found by [18] in crossbred goats SRD×Boer (2.10) and SRD×Anglonubian (2.20). Despite these authors having assigned a slightly larger value directly to the carcass, which reinforces the evidence that intramuscular fat condition observed in this study has the potential to meet the market demands, considering that goats are seen as animal lean meat.

The superiority when comparing to the goats values to other genotypes, focusing on meat production, is explained by the case that the animals were intended for breeding were kept under more appropriate management. However, such superiority was also influenced by age, since animals aged 7 months were evaluated. After this age there is the possibility of increased deposition of fat in the carcass [19].

The good body condition and potential of carcass marbling of the animals in study are emphasized when compared to results obtained in sheep. For MRE, the average value was 2.55 points, close to that observed in crossbred undefined sheep breed (SRD)×Santa Inês (2.98), which did not differ statistically from crossbred lambs SRD×Texel [20]. Thus, due the lack of studies to assess these traits in goats, the observed values in this study are close to those observed in sheep [21].

Based on the Monte Carlo error (MCE), low values for both the analyses with linear and threshold models confirm the convergence, because according to [16], this occurs when the error value is added to the average estimate of distribution *posteriori* heritability coefficient, it does not change the value of this estimate, considering the second decimal place, as shown in Table 2.

Therefore, the convergence monitored by Geweke criterion and MCE indicated that the chain size used in this study was appropriate and the amount *a posteriori* were heritability valid estimates of categorical traits.

There is tendency of BF to be more sensitive to the choice of the prior distribution than the *a posteriori* interval probability [14]. Thus, the threshold model was presented as the greater one with ability to detect the genetic variability in MRE and BCS, when compared to the linear model, according to [3], related to the efficiency of this model for categorical data.

Even considering that in the literature there are few studies that compare linear and threshold models for analysis of categorical carcass traits in goats, our results agree with the consulted studies, mostly with cattle and sheep, indicating that the use of threshold model provides more accurate estimates than the linear model [22,23]. However, studies have not verified differences among models [24].

The lower variability of the genetic components of categorical traits, when estimated by linear model, could be an evidence of the inadequacy of its use for traits with this distribution, which may result in estimates that cause wrong inferences [22]. Reflections of this are that estimates of heritability for BCS and MRE traits may be overestimated using this model, leading to genetic progress estimation with low accuracy.

It was found that the use of the threshold model resulted in lower estimates for obtaining the studied heritability of categorical traits, because the residual variance estimates differed markedly between threshold and linear models. In addition, as the credibility regions do not overlap, this leads to rejection of statistical hypothesis of equality between the estimates generated by the two models [1].

As seen in the single-trait analysis, it was found that the Geweke convergence criterion presented significance level lower than 0.05, and MCE was low for all analyses (Table 3), implying that the Bayesian analysis with linear threshold model was adequate for obtaining *a posteriori* distribution estimates of the parameters in two-trait analyses.

It was found in two-trait analyses increased contribution of the considered information number and also influence of the correlations between the characteristics to increase the estimated values for the components of (co) variance and heritability values of the traits in study (Table 3), since there was recovery of part of the additive genetic variance that was incorporated into the residual variance in single-trait analysis (Table 2).

Adipose tissue is the component of the carcass which has the highest variability and influence of the environment [25], which might also be inherent in the MRE behavior that is intramuscular fat located in the *longissimus dorsi*. Thus, it is considered that this fact is an explanation for the low heritability estimates for MRE, and then much of the phenotypic variability of the trait is explained by environmental component, so it is important to pay attention to the environmental factors that influence the phenotypic expression of marbling.

The consulted literature was incipient to the heritability estimates of BCS and carcass marbling in goats. However, the heritability values of the traits MRE and BCS equal to 0.13 and 0.09, respectively. These values higher than the univariate analysis with threshold model, are of low magnitude, then show little potential response to selection based on these characteristics in the evaluated breed.

So, there is low possibility for response to direct selection or by including these traits in selection indexes. Therefore, considering the producer interest in higher meat production and carcass yield, the index should prioritize animal selection with higher genetic value in REA, if the interest is the finishing precocity the FTS gains importance.

These categorical traits should not be considered solely in meat goat breeding programs, because besides the amount of meat, the carcass needs adipose protection for cooling, which is guaranteed by the presence of subcutaneous fat, additionally it can be considered an early indicator of development [26]. Therefore, both REA and FTS should be considered in meat goats breeding programs and the heritability coefficient estimates of 0.28 and 0.24, respectively, both have potential to make beef goats selection indexes.

It is observed in Table 4 that MRE showed moderate genetic association with BCS, so there are common genes responsible for the expression of these traits. Therefore, there is potential for genetic gain by selecting one of them as a phenotypic marker, or both do not necessarily need to be in the same selection index. Thus, it is evident the importance of identifying correlated response when selection is based on a trait and the intention is also to improve another one, which is defined by the genetic correlation between them, but also influences the heritability of traits involved [27].

Identical situation was observed in the genetic correlation between FTS and MRE (0.58). So, considering the ease of measuring the thickness of subcutaneous fat in the sternal region, it is indication useful means of indirect selection for marbling. The heritability of FTS being 0.24 suggests the inclusion of this trait in the selection indexes for carcass production with good grades of marbling.

Thus, BCS and FTS would be more suitable to be included in the composition of selection indexes, since they have higher heritability, but with equal values to 0.13 and 0.24, respectively, which are considered low values for direct selection purposes (Table 3). However, as the genetic correlations between these traits and marbling were equal to 0.58 (Table 4), this indicates that there is an increasing trend in the carcass marbling.

However, BCS and FTS did not appear to be genetically correlated with each other, which was unexpected and fails to explain the presence of genetic correlation of moderate amplitude of both to the marbling. In other words due to existence of genes in common. However, it is relevant to consider that these traits are measured almost directly in the *longissimus dorsi*.

Considering that the estimated genetic correlation between MRE and HH was equal to 0.88 (Table 4) and the estimated heritability for HH was 0.32 (Table 3), it is a good indication that the selection based on HH can provide, indirectly, higher genetic progress in the carcass marbling. In this case the HH could be a potential alternative of a phenotypic marker for carcass marbling improvement. This has also the advantage of high heritability, combined with ease of measurement at low cost. However, it can be considered that taller animals grow faster and tend to have less fat [28].

The genetic association of MRE with REA was small (0.03), implying low potential response correlated by selection based on the other one. As REA showed heritability equals to 0.28, considered moderate magnitude and response potential to direct selection, which was also found in cattle. It is recommended to compose selection indexes in the breed and it will not interfere in carcass marbling. The deposition profile of these tissues in the carcass may be related to this result, because as the muscle is earlier developed than the adipose tissue, which implies in low correlation between these characteristics [28].

It is also observed that the genetic correlation between body weight and loin marbling had low magnitude, showing that heavy animals and, consequently, with better body condition, do not necessarily present greater amount of fat in the carcass. This result can be seen as an indication that goats present the “lean meat animal” profile, which has the disadvantage of limiting cryopreservation. On the other hand, it would meet market demands of low-fat products.

## IMPLICATIONS

Heritability estimates for categorical traits of BCS and carcass marbling using ultrasound were from low to moderate magnitude, so genetic progress is possible with animal selection based on these characteristics. The two-trait analysis provided higher estimates of heritability for the traits in study. The threshold model showed best adjustment for categorical characteristics, BCS, and carcass marbling using ultrasound, with overestimation trend of heritability when the data were submitted to analysis under the linear model. Direct selection of the continuous distribution of characteristics, such as thickness sternal fat and HH, allows obtaining the indirect selection for ribeye marbling.

## Figures and Tables

**Table 1:** Descriptive statistics of categorical traits of marbling ribeye (MRE) and body condition score (BCS), in Anglonubian goats

Trait	Average	Median	Mode	SD	VC	s^2^
MRE	2.55	3.00	3.00	1.16	45.71	1.36
BCS	3.27	3.00	3.00	1.11	34.11	1.24

SD, standard deviation; VC, variation coefficient; s^2^, variance.

**Table 2:** Additive variance estimates and heritability (h^2^) of categorical characteristics body condition score (BCS) and the loin marbling (MRE), obtained from analysis with linear model and threshold model

Trait	Model	$\sigma_{a}^{2}$	*h* ^2^	Geweke (p-value)	MCE	CI	DIC	BF
BCS (grade 1 to 5)	Linear	0.20	0.29	0.04	0.002	0.00–0.36	956.44	0.45
	Threshold	2.23	0.11	0.03	0.001	0.00–0.14	412.00*	
MRE (grade 1 to 6)	Linear	0.12	0.12	0.00	0.001	0.00–0.23	1,176.10	0.62
	Threshold	0.11	0.03	0.00	0.000	0.00–0.04	706.74*	

$\sigma_{a}^{2}$, additive genetic variance; MCE, Monte Carlo error; CI, confidence interval of heritability; DIC, information criterion of deviance; BF, bayes factor.

* Best adjustment model.

**Table 3:** Estimates of the (co) variance and heritability (h^2^) components of body condition score characteristics (BCS), the loin marbling (MRE), ribeye area (REA), fat thickness of the sternum (FTS), hip height (HH), the leg perimeter (LP) and body weight, obtained in two-trait analysis

Characteristic	$\sigma_{a}^{2}$	$\sigma_{e}^{2}$	*h* ^2^	Geweke (p-value)	MCE	IC
BCS (grade 1 to 5)	3.48	22.71	0.13	0.00	0.001	0.00–0.17
MRE (grade 1 to 6)	0.40	3.81	0.09	0.03	0.000	0.00–0.08
REA (cm^2^)	0.79	2.03	0.28	0.01	0.002	0.03–0.37
FTS (mm)	0.55	1.77	0.24	0.00	0.001	0.03–0.34
HH (cm)	3.91	8.29	0.32	0.03	0.002	0.07–0.41
LP (cm)	6.75	9.55	0.41	0.02	0.003	0.16–0.47
Body weight (kg)	62.23	52.78	0.54	0.00	0.003	0.51–0.55

$\sigma_{a}^{2}$, additive genetic variance; $\sigma_{e}^{2}$, residual variance; MCE, Monte Carlo error; IC, confidence interval of heritability.

**Table 4:** Genetic correlations of categorical traits of marbling ribeye (MRE) and body condition score (BCS) with ribeye area, fat thickness sternal, hip height, leg perimeter, and body weight

Continuous distribution traits	MRE	BCS
Body condition score	0.58	-
Ribeye area	0.03	0.32
Fat thickness sternal	0.58	0.04
Hip height	0.88	0.00
Leg perimeter	0.03	0.09
Body weight	0.13	0.30

## References

[1] Faria CU, Magnabosco CU, Albuquerque LG (2008). Genetic analysis for visual scores of bovines with the linear and threshold bayesian models. Pesq Agropec Bras.

[2] Gianola D, Foulley JL (1983). Sire evaluation for ordered categorical data with a threshold model. Gen Sel Evol.

[3] Luo MF, Boettcher PJ, Schaeffer LR, Dekkers JCM (2002). Estimation of genetic parameters of calving ease in first and second parities of Canadian Holsteins using Bayesian methods. Livest Prod Sci.

[4] Misztal I (1989). Programs for analysis of mixed linear and threshold models with support for REML- type variance component estimation and maternal grandsire model. CMMAT2.

[5] Sorensen DA, Andersen S, Gianola D, Korsgaard I (1995). Bayesian inference in threshold models using Gibbs sampling. Genet Sel Evol.

[6] Van Tassell CP, Van Vleck LD, Gregory KE (1998). Bayesian analysis of twinning and ovulation rates using a multiple-trait threshold model and Gibbs sampling. J Anim Sci.

[7] Ribeiro SDA (1998). Rational breeding of goats.

[8] United States Department of Agriculture (USDA) (1997). Standards for grades of Slaughter cattle and standards for grades of carcass beef. Agricultural Marketing Services, USDA.

[9] Figueiredo Filho LAS, Sarmento JLR, Campelo JEG, Santos NPS, Sousa A (2012). Measures Carcass traits by ultrasound in goats. Rev Bras Saúde Prod Anim.

[10] Misztal J (2002). Fortran programs [Internet].

[11] Geweke J (1992). Evaluating the accuracy of sampling-based approaches to calculating posterior moments. Bayesian Statistics.

[12] Sorensen DA, Gianola D (2002). Likelihood, Bayesian and MCMC methods in quantitative genetics: statistics for biology and health.

[13] Spiegelhalter DJ, Best NG, Carlin BP, Van der Linde A (2002). Bayesian measures of model complexity and fit. J R Statist Soc B.

[14] Kass RE, Raftery AE (1995). Bayes factors. J Am Statist Assoc.

[15] Dempster AP (1997). The direct use of likelihood for significance testing. Statist Comput.

[16] Van Tassell CP, Van Vleck LD (1996). Multiple-trait Gibbs sampler for animal models: flexible programs for Bayesian and likelihood-based (co)variance component inference. J Anim Sci.

[17] Figueiredo Filho LA, Do ÓA, Sarmento JLR, Santos NPS, Torres TS (2015). Genetic parameters for carcass traits and body size in sheep for meat production. Trop Anim Health Prod.

[18] Brito EA, Sousa WH, Ramos JPF, Oliveira S (2009). Qualitative features of the carcass of three genetic groups of goats and sheep finished in confinement. Rev Tecnol Ciên Agropec.

[19] Hammond J (1965). Farm animals: their breeding, growth, and inheritance.

[20] Suguisawa L, Vargas FM, Marques ACW Carcass characteristics and meat quality by ultrasonography in confined lamps.

[21] Bonacina MS, Osório MTM, Osório JCS, Corrêa GF, Hashimoto JH (2011). The influence of sex and finishing system on carcass and meat quality of Texel × Corriedale lambs. R Bras Zootec.

[22] Santos NPS, Sarmento JLR, Pimenta Filho EC (2013). Environmental and genetic aspects of litter size in goats using linear and threshold bayesian models. Arq Bras Med Vet Zootec.

[23] Fernandes PB, Santana LA, Ferreira FR Use of linear and threshold models for the estimation of variance components for pregnant probability from Murrah buffaloes.

[24] Faria CU, Andrade WBF, Pereira CF, Silva RP, Lôbo RB (2015). Bayesian analysis for carcass traits in Polled Nelore. Cienc Rural.

[25] Rosa GT, Pires CC, Silva JHS, Motta OS (2005). Muscle, fat and bone allometric growth in Texel lambs carcasses cuts in relation to the feeding methods and slaughter weight. Cienc Rural.

[26] Koury Filho W, Albuquerque LG, Forni S (2010). Genetic parameters estimates for visual scores and their association with body weight in beef cattle. R Bras Zootec.

[27] Falconer DS (1987). Introduction to quantitative genetics.

[28] Sainz RD, Araujo FRC Types of cattle of bovine and swine.

